# Supplementary material for: Treatment of High-Risk Neuroblastoma with Dinutuximab and Chemotherapy Administered in all Cycles of Induction
Source: Cancers (Basel). 2023 Sep 18;15(18):4609. doi: 10.3390/cancers15184609 (PMC10527563; doi:10.3390/cancers15184609)
Supplement: Supplementary file 1 [file cancers-15-04609-s001.zip › cancers-2549432-supplementary.pdf]

cancers

MDPI

Supplementary Materials

Supplementary Figure S1. Flow diagram of depicting the treatment options for newly diagnosed HR NBL who were treated at SJCRH for between 2017 and 2022.

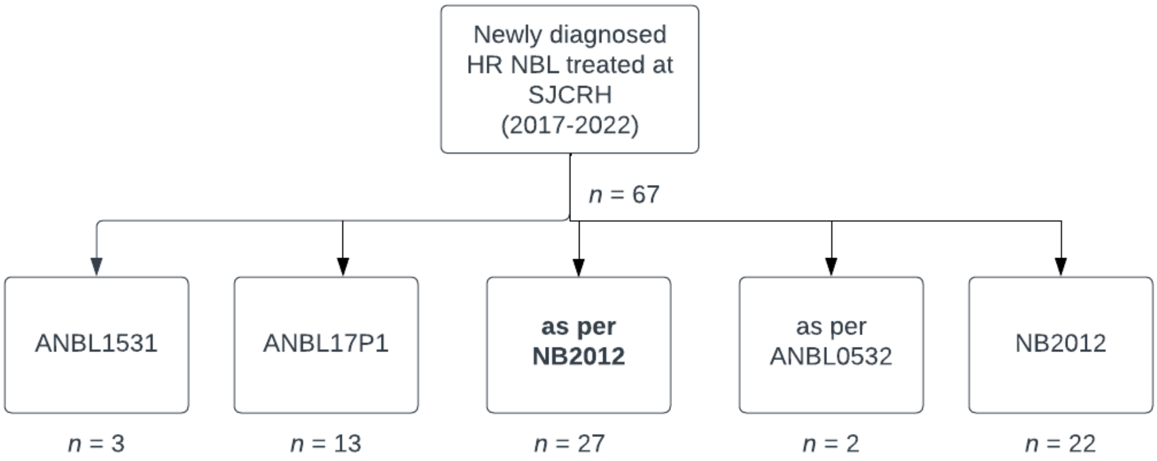

Supplementary Figure S2. Schema of the administered treatment (with key).

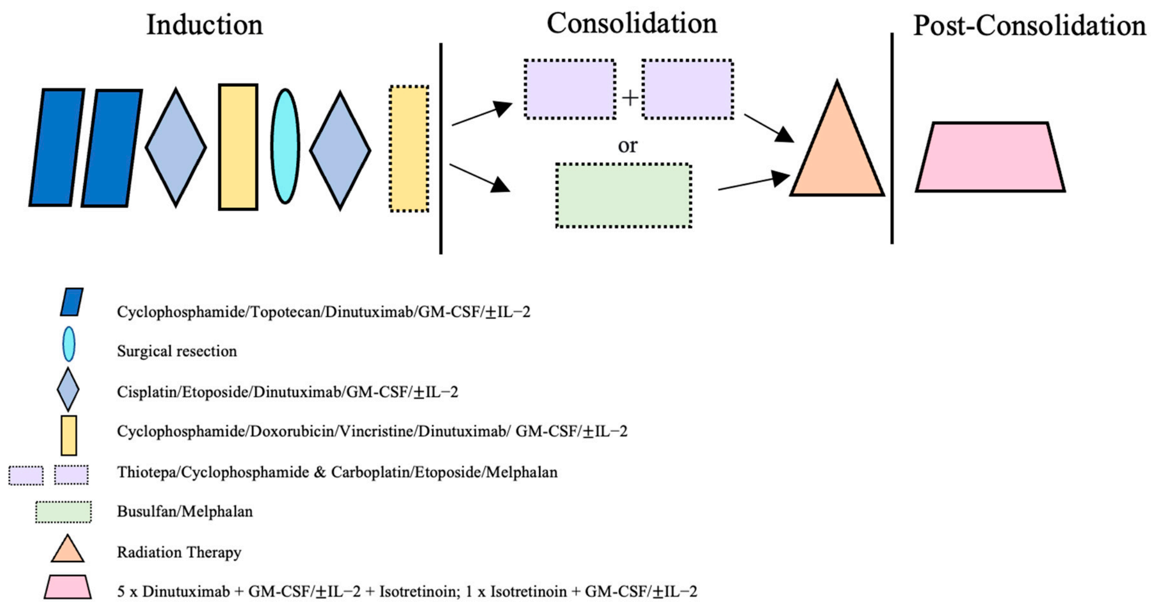

Abbreviations: RT: Radiation therapy, Granulocyte-macrophage colony-stimulating factor (GM-CSF), Interleukin-2 (IL-2). Note: During Induction, GM-CSF was given at 250 mcg/m^2/day from day 6 of each cycle until ANC ≥ 2000/mm^3 or until ANC ≥ 500/mm^3 AND cycle day 21. Surgical resection completed at the discretion of the surgeon between cycles 3 and 5.

Supplementary Table S1. Treatment Plan/Dosages with 5-6 Cycles of Induction Chemotherapy

Cycle	Week	Drug and Dosages
1	0	Cyclophosphamide:<br>**>12 kg:** 400 mg/m²/dose IV days 1-5<br>**≤12 kg:** 13.3 mg/kg/dose IV days 1-5<br>Topotecan (TPT): 1.2 mg/m²/day IV days 1-5<br>Dinutuximab 17.5 mg/m² IV days 2-5<br>± Low-dose IL-2 SQ 1 million units/m² for 6 doses (days 6, 8, 10, 12, 14, and 16)<br>SQ GM-CSF begun on day 6 at 250 mcg/m²/day, until a post-nadir ANC ≥ 2000/mm³
2#	3	Cyclophosphamide:<br>**>12 kg:** 400 mg/m²/dose IV days 1-5<br>**≤12 kg:** 13.3 mg/kg/dose IV days 1-5<br>Topotecan (TPT): 1.2 mg/m²/day IV days 1-5<br>Dinutuximab 17.5 mg/m² IV days 2-5<br>± Low-dose IL-2 SQ 1 million units/m² for 6 doses (days 6, 8, 10, 12, 14, and 16)<br>SQ GM-CSF begun on day 6 at 250 mcg/m²/day, until a post-nadir ANC ≥ 2000/mm³
**Evaluate response/PBSCH/ Resect tumor if possible**[α]		
3	6	CISplatin:<br>**>12 kg:** 50 mg/m²/day x 4 IV over 1 hour (days 1-4)<br>**≤12 kg:** 1.66 mg/kg/day x4 IV over 1 hour (days 1-4)<br>Etoposide[@]:<br>**>12 kg:** 200 mg/m²/day x 3 IV over 1 hour (days 1-3)<br>**≤12 kg:** 6.67 mg/kg/day x3 IV over 1 hour (days 1-3)<br>Dinutuximab 17.5 mg/m² I.V days 2-5<br>± Low-dose IL-2 SQ 1 million units/m² for 6 doses (days 6, 8, 10, 12, 14, and 16)<br>SQ GM-CSF begun on day 6 at 250 mcg/m²/day, until a post-nadir ANC ≥ 2000/mm³
4	9	Cyclophosphamide:<br>**>12 kg:** 2.1 gm/m² daily x 2 IV days 1 and 2<br>**≤12 kg:** 70 mg/kg/day x2 IV days 1 and 2<br>DOXOrubicin:<br>**>12 kg:** 25 mg/m² IV intermittent infusion days 1 – 3<br>**≤12 kg:** 0.83 mg/kg/day IV intermittent infusion days 1-3<br>MESNA:<br>**>12 kg:** 525 mg/m² IV immediately prior to each cyclophosphamide infusion and again at 3, 6 and 9 hours after each cyclophosphamide<br>**≤12 kg:** 17.5 mg/kg/dose IV immediately prior to each cyclophosphamide infusion and again at 3, 6 and 9 hours after each cyclophosphamide<br>VinCRIStine^:<br>**<12 months**: 0.017 mg/kg/dose IV daily x3 days<br>**≥ 12 months:** 0.67 mg/m2/dose or 0.022 mg/kg/dose-whichever is lower IV daily x3 days (max 0.67 mg/dose)<br>**≥12 months and ≤12 kg**: 0.022 mg/kg/dose IV daily x3 days<br>Dinutuximab 17.5 mg/m² I.V days 2-5<br>± Low-dose IL-2 SQ 1 million units/m² for 6 doses (days 6, 8, 10, 12, 14, and 16)<br>SQ GM-CSF begun on day 6 at 250 mcg/m²/day, until a post-nadir ANC ≥ 2000/mm³
5	12	CISplatin:<br>**>12 kg:** 50 mg/m²/day x 4 IV over 1 hour (days 1-4)<br>**≤12 kg:** 1.66 mg/kg/day x4 IV over 1 hour (days 1-4)<br>Etoposide[@]:

		**>12 kg:** 200 mg/m^2/day x 3 IV over 1 hour (days 1-3)<br>**≤12 kg:** 6.67 mg/kg/day x3 IV over 1 hour (days 1-3)<br>Dinutuximab 17.5 mg/m^2 I.V days 2-5<br>± Low-dose IL-2 SQ 1 million units/m^2 for 6 doses (days 6, 8, 10, 12, 14, and 16)<br>SQ GM-CSF begun on day 6 at 250 mcg/m^2/day, until a post-nadir ANC ≥ 2000/mm^3
±6	15	Cyclophosphamide:<br>**>12 kg:** 2.1 gm/m^2 daily x 2 IV days 1 and 2<br>**≤12 kg:** 70 mg/kg/day x2 IV days 1 and 2<br>DOXOrubicin:<br>**>12 kg:** 25 mg/m^2 IV intermittent infusion days 1 – 3<br>**≤12 kg:** 0.83 mg/kg/day IV intermittent infusion days 1-3<br>MESNA:<br>**>12 kg:** 525 mg/m^2 IV immediately prior to each cyclophosphamide infusion and again at 3, 6 and 9 hours after each cyclophosphamide<br>**≤12 kg:** 17.5 mg/kg/dose IV immediately prior to each cyclophosphamide infusion and again at 3, 6 and 9 hours after each cyclophosphamide<br>VinCRIStine^:<br>**<12 months**: 0.017 mg/kg/dose IV daily x3 days<br>**≥ 12 months:** 0.67 mg/m2/dose or 0.022 mg/kg/dose-whichever is lower IV daily x3 days (max 0.67 mg/dose)<br>**≥12 months and ≤12 kg**: 0.022 mg/kg/dose IV daily x3 days<br>Dinutuximab 17.5 mg/m^2 I.V days 2-5<br>± Low-dose IL-2 SQ 1 million units/m^2 for 6 doses (days 6, 8, 10, 12, 14, and 16)<br>SQ GM-CSF begun on day 6 at 250 mcg/m^2/day, until a post-nadir ANC ≥ 2000/mm^3

[α] Primary tumors were resected when deemed feasible by our surgeons.

[@]Was substituted for etoposide phosphate (etopophos) if patients reacted to etoposide.

^ Total dose of vincristine did not exceed 2 mg in 72 hours or 0.67 mg/day for any patient.

#In addition to GM-CSF, patients received daily subcutaneous granulocyte-colony stimulating factor (G-CSF) at 5 mcg/kg in preparation of stem cell harvest.

± Included for some patients only.

Abbreviations: SQ-subcutaneous, GM-CSF: Granulocyte macrophage colony-stimulating factor, PBSCH: peripheral blood stem cell harvest

Supplementary Table S2. Patient Characteristics

**Characteristic**	**(n=27) No. (%)**
**Age at diagnosis, months**	
<18	5 (19)
≥ 18	22 (81)
**Disease status before Induction**	
Newly-diagnosed	23 (85)
Recurrent/refractory after IR therapy	3 (11)
Recurrent after surgery alone	1 (4)
**Sex**	
Male	11(41)
Female	16 (59)
**Race**	
White	16 (59)
Black	7 (26)
Other	4 (15)
**INRG Stage**	
M	26 (96)
L2	1 (4)
***MYCN* status**	
Amplified	11 (41)
Non-amplified	16 (59)
**Shimada histology**	
Favorable	4 (15)
Unfavorable	15 (55)
Unknown	8 (30)
**Primary tumor site**	
Retroperitoneum	3 (11)
Adrenal gland	22 (81)
Bilateral adrenal glands	1 (4)
Bone marrow	1 (4)
**Sites of metastatic disease**	
Bone	23 (85)
Bone marrow	21 (78)
Lymph node	12 (44)
Liver	4 (15)
Lung/pleura	3 (11)
**Segmental chromosomal aberrations**	
17q gain	8 (30)
11q LOH	2 (7)
1p LOH	0 (0)
Unknown	15 (55)

Abbreviations: IR: intermediate risk, INRG: The International Neuroblastoma Risk Group[6] LOH: Loss of heterozygosity
